# Analysis of Crack Propagation Behaviors in RPV Dissimilar Metal Welded Joints Affected by Residual Stress

**DOI:** 10.3390/ma16196578

**Published:** 2023-10-06

**Authors:** Lingyan Zhao, Yuchun Sun, Zheren Shi, Bin Yang

**Affiliations:** 1 School of Science, Xi’an University of Science and Technology, Xi’an 710054, China; 2 School of Mechanical Engineering, Xi’an University of Science and Technology, Xi’an 710054, China; sunyuchun@stu.xust.edu.cn (Y.S.); 22205224093@stu.xust.edu.cn (B.Y.); 3 AVIC Qing’an Group Co., Ltd., Xi’an 710077, China; 20205224072@stu.xust.edu.cn

**Keywords:** residual stress, dissimilar metal welded joints, crack propagation, XFEM, yield strength

## Abstract

In severe service environments, the presence of high local residual stress, significant organizational gradient, and nonlinear changes in material properties often leads to stress corrosion cracking (SCC) in dissimilar metal welded (DMW) joints. To accurately predict the crack growth rate, researching the initiation and propagation behavior of SCC cracks in DMW joints under residual stress (RS) is one of the most important methods to ensure the safe operation of nuclear power plants. Using the extended finite element method (XFEM), the crack propagation behaviors in DMW joints under different RS states are predicted and compared. The effects of RS, crack location, and initial crack length on crack propagation behavior are investigated. The crack in a DMW joint without RS deflects to the material of low yield strength. High residual stress urges the crack growing direction to deflect toward the material of high yield strength. Young’s modulus has little impact on the crack deflection paths. The distance between the specimen symmetric line and the boundary line has little effect on the crack initiation and propagation within the RS field. A long initial crack is more likely to initiate and propagate than a short crack. To a long crack and the crack that is far from the interface of two materials, the impact of residual stress on the crack propagation path is significant when it is located in a material with high yield strength, while when the initial crack is located in the material with low yield strength, RS has a great influence on the deflection of a short crack growth direction on the condition that the crack is adjacent to the interface.

## 1. Introduction

Welding is a key process in the manufacturing of nuclear power pressure vessels and pipelines. Steam generators (SG) and the reactor pressure vessels (RPV) contain a large number of homogeneous and dissimilar metal welded (DMW) joints, whose materials have good mechanical and corrosion resistance, such as austenitic stainless steel and Ni-base alloys [1,2]. These similar and dissimilar welded joints are usually made by conventional arc welding methods such as gas metal arc welding (GMAW) and gas tungsten arc welding (GTAW) [3,4,5]. High local stresses, large organizational gradients, and materials properties with nonlinear gradient changes induced by welding have a certain impact on the stress corrosion resistance of materials and the initiation and propagation mechanism of cracks that are located in a DMW joint [6,7,8]. In harsh service environments, residual stress (RS) is one of the important factors that lead to the problems of stress corrosion cracking (SCC) [9,10,11] and intergranular corrosion cracking (IGSCC) [12,13]. It poses a serious threat to the long-term safe operation of welding structures in nuclear power plants. To accurately predict the crack growth rate, efforts to understand the initiation and propagation behaviors of SCC cracks in DMW joints under RS are one of the most important means to ensure the safe operation of nuclear power plants.

The combination of high hardness, stress concentration, and unevenly distributed high residual strain in the region between the fusion boundary and the weld metal can result in high-stress corrosion sensitivity [14,15]. The accumulation of plastic strain around the crack tip leads to the release and redistribution of RS. Residual stress concentration at the weld edge may be the main cause of crack initiation and propagation [16]. Although post-weld heat treatment can reduce welding-induced RS, it does not reduce the SCC growth rate in high-temperature water environments [17]. This suggests that the relationship and mutual influencing mechanisms between residual stress and the driving force for propagation remain unclear.

Due to the complex geometric dimensions, microstructure, load levels, and multi-axis state of actual components, it is difficult to analyze the impact of RS on actual components. Therefore, small laboratory specimens are usually investigated under closely controlled laboratory conditions and using the finite element method. A tensile opening RS field was introduced in the vicinity of the notched compact tension (CT) specimens by using the in-of-plane pre-compression method. A good correlation was found between measurements and finite element predictions of strain and stress before and after thermal exposure [18]. In later research, it was shown that a tensile RS field ahead of a notch can be generated by loading in compression beyond yield and then unloading. Moreover, the extent of the tensile RS field can be changed by altering the loading point displacement, notch radius, and *a*/*W* ratio of the CT specimens. Research demonstrated that creep strain and damage accumulate ahead of notches during RS relaxation [19]. CT specimens with tensile RS and externally applied stress exhibited a fast creep crack growth tendency compared with CT specimens with only externally applied stress [20]. Tensile stress promotes crack elongation, while compressive stress can delay crack initiation and impede crack propagation. However, the growth rate of creep crack remains largely unaffected by RS [21,22]. The longitudinal RS is subjected to tension in the thickness direction of the steel plate, resulting in a higher stress intensity factor (SIF) than that of the steel plate without RS. When the crack tip is located within the compressive stress zone, the external load leads to a reduction in SIF [23].

The application and secondary development of finite element (FE) numerical simulation method, especially the extended finite element method (XFEM), made it possible to simulate the crack propagation path and growing direction under the interaction of uneven strength of materials performance and RS [24,25,26]. The initial position, length, and complex fluctuating loads of cracks result in different fracture resistance and crack propagation paths [27,28]. The crack deflects to the low yield strength side when it is located in a mechanical field with an uneven yield strength distribution. The crack length of welded joints, as well as the plastic deformation range of the crack tip in high-stress areas can be reduced with the increase in yield strength along the crack growing direction [29]. When the initial crack parallels the fusion boundary (FB) line, the crack will deviate from it. The crack propagation length is smaller as the initial crack tip is closer to the FB line when the load condition is constant [30]. A longer initial crack is more easily extended. The residual stress, the thickness of the residual stress layer, and the initial crack length have significant influences on the crack growth. However, it is essential to note that the RS layer should not be overly thick [31].

During the process of crack propagation, the stress–strain field and RS field at the crack tip interact. The accumulation of plastic strain at the crack tip can result in the release and redistribution of RS. However, there is limited discussion regarding crack propagation in DMW joints subjected to RS or redistributed RS. In this study, specimens of DMW joints under RS and applied loads were considered. The XFEM technique was employed to simulate the propagation behavior of cracks located at the interfaces of different materials. The study examines the influence of RS, Young’s modulus, crack location, and their initial lengths on crack propagation paths and growth directions, offering a novel approach for accurately assessing crack propagation behavior driven by RS in DMW joints.

## 2. Damage Model in XFEM

To depict and simulate crack propagation in XFEM, it is crucial to integrate a crack initiation criterion and a crack propagation law to establish the damage model. The primary characteristic parameter for defining the crack initiation criterion is the maximum principal stress, denoted as *σ*_max_ at the crack tip. In this simulation, the selected crack initiation criterion is based on the maximum principal stress. The ratio of the maximum principal stress is defined as *f* [32]:

(1) $$f=\left\{\frac{\langle \sigma_{\max}\rangle }{\sigma_{\max}^{0}}\right\}$$

(2) $$\langle \sigma_{\max}\rangle =\left\{\begin{array}{c} 0,\\ \sigma_{\max}, \end{array}\right.\begin{array}{c} \sigma_{\max}<0\\ \sigma_{\max}\geq 0 \end{array}$$

In this context, $\sigma_{\max}^{0}$ represents the maximum allowable principal stress. The Macaulay brackets 〈 〉 indicate that crack initiation does not occur under purely compressive stress conditions. The assumption is that the crack initiates when the ratio *f* reaches one.

Once the crack begins to initiate, the behavior of crack propagation is characterized by the equivalent fracture energy release rate, denoted as *G_equiv_*. Crack initiation occurs when *G_equiv_* reaches the critical equivalent fracture energy release rate, referred to as *G_equivC_*. To represent the fracture resistance of the material, critical energy release rates for Mode I, Mode II, and Mode III cracks are introduced, denoted as *G*_І_, *G*_П_, and *G*_Ш_, respectively.

(3) $$\frac{G_{\mathrm{equiv}}}{G_{\mathrm{equiv}}}={\left(\frac{G_{Ι}}{G_{ΙC}}\right)}^{a_{m}}+{\left(\frac{G_{ΙΙ}}{G_{ΙΙC}}\right)}^{a_{n}}+{\left(\frac{G_{ΙΙΙ}}{G_{ΙΙΙC}}\right)}^{a_{0}}$$

where *a*_m_, *a*_n_, and *a*_0_ are damage exponents.

The energy release rate, *G*, represents the force required for the unit length of crack propagation and serves as the prime driving force for crack propagation.

(4) $$G=-\frac{dΙΙ}{dA}=-\underset{\Delta A\to 0}{\lim}\frac{\Delta ΙΙ}{\Delta A}=-\underset{\Delta A\to 0}{\lim}\frac{\Delta ΙΙ}{B\Delta a}$$

where П = *U*−*M* is potential energy; Δ*A* is cracked surface area; *U* is strain energy of the crack; *M* is external work; and *a* and *B* are the crack length and crack width, respectively.

For an approximate calculation of the fracture energy release rate *G*, a sufficiently small increment Δ*a*, approaching zero is specified,

(5) $$G=\frac{Ι{Ι}_{2}-Ι{Ι}_{1}}{B\Delta a}$$

where the potential energy П_1_ in step 1 with the crack length a can be calculated by П_1_ = *U*_1_−*W*_1_, and the potential energy П_2_ in step 2 with the crack length *a* + Δ*a* can be calculated by П_2_ = *U*_2_−*W*_2_.

## 3. Finite Element Model of Cracks in DMW Joints

### 3.1. Geometric Model

The manufacturing process for a nozzle/safe-end joint comprises several thermal procedures, including cladding, buttering, post-weld heat treatment (PWHT), and dissimilar metal multi-pass welding. A tungsten inert gas (TIG) arc welding method was used to conduct cladding, buttering, and dissimilar metal multi-pass welding, whose corresponding process parameters during the processes are the same, welding current 120–160 A, voltage 10–12 V, and welding speed 120–200 mm/min. Then, the PWHT was used to reduce residual stress and improve the toughness in the heat-affected zone. The temperature and the time of heat treatment are 600 ± 20 °C for 7 h.

**Figure 1:**
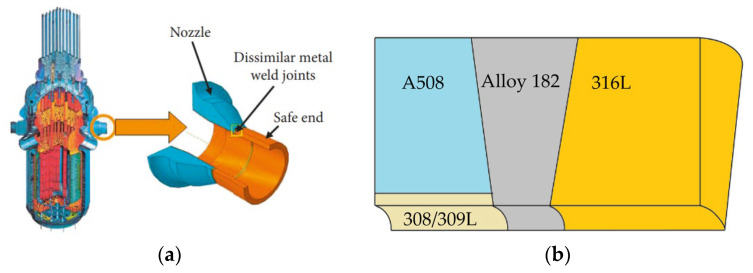
The DMW joint to connect an RPV nozzle and a safe-end pipe: (**a**) the location and (**b**) the material composition.

**Figure 2:**
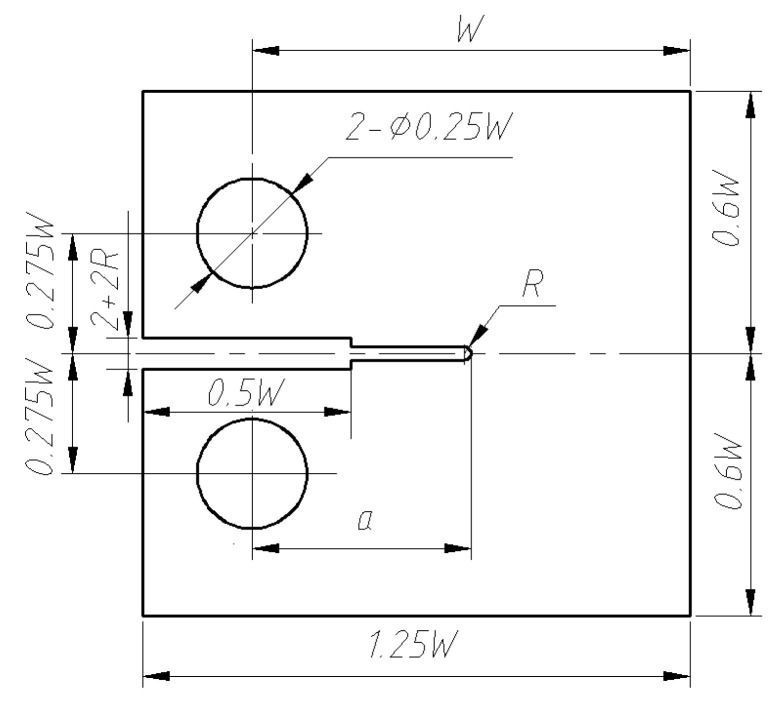
Geometry size of 1T-CT specimen.

A typical DMW joint, which connects an RPV nozzle and a safe-end pipe, can be simplified as a pipe. The dimensions of its inner diameter and wall thickness are both significantly larger than the crack length. Thus, the problem of crack propagation in DMW joints can be simplified as a plane strain problem. In accordance with ASTM E399-90 [33], a one-inch compact tension (1T-CT) specimen was modeled. The specimen was taken from the DMW joint, as shown in Figure 1. The geometrical shape and dimensions of the 1T-CT specimen are shown in Figure 2, where *W* = 50 mm, *a* = 0.5 W, and *R* = 0.25 mm.

### 3.2. Material Model

Materials used in pressurized water reactor DMW joints typically exhibit power-hardening behavior. This material is characterized by a nonlinear relationship between stress and strain, which can be mathematically described by the Ramberg–Osgood equation:

(6) $$\frac{\varepsilon }{\varepsilon_{0}}=\frac{\sigma }{\sigma_{0}}+\alpha {\left(\frac{\sigma }{\sigma_{0}}\right)}^{n}$$

where *α* is the material offset coefficient, *σ* is true stress, *σ*_0_ is yield strength, *ε* is true strain, and *ε*_0_ is yield strain.

The hardening exponent *n* can be expressed as

(7) $$n=\frac{1}{\kappa \ln(1390/\sigma_{0})}$$

where *κ* = 0.163 [34].

In the creation of a DMW joint, A508 and 316L were employed as the base metal, while Alloy 182 served as the welding metal. The pertinent constitutive parameters of the materials used in the calculation are listed in Table 1.

### 3.3. Mesh Model

**Figure 3:**
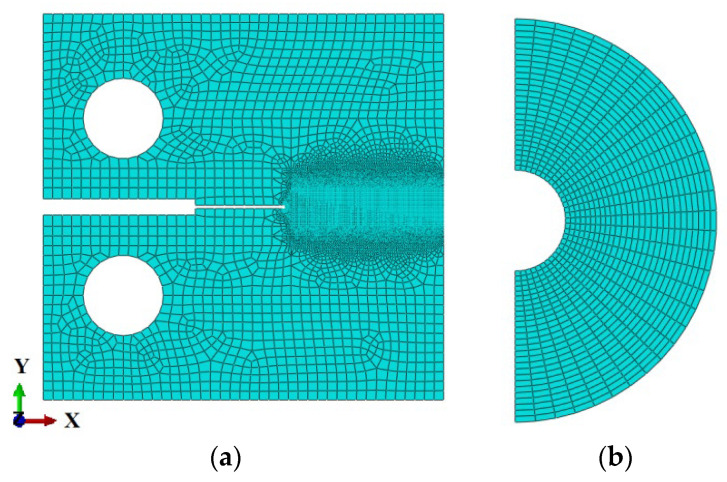
Mesh model: (**a**) the global mesh and (**b**) the mesh at the crack front.

Because XFEM employs a unique displacement function that addresses the issue of discontinuity and achieves independence between the discontinuous interface and the mesh, a structured quadrilateral mesh is exclusively utilized in the crack extension region and the weld zone. A free quadrilateral mesh is employed elsewhere to eliminate inaccuracies caused by the hourglass phenomenon. A circular area with a radius of 2 mm is subdivided around the crack tip to create a finer mesh, ensuring the accuracy of the crack tip initiation location. The entire model comprises 49,619 elements, composed of 4-node biquadratic plane strain quadrilaterals (CPE4), as illustrated in Figure 3.

The accuracy of numerical simulations is notably affected by the element size near the crack tip. To guarantee precision, the element size *h* in the crack propagation region should meet the criteria specified in Equation (8) [37].

(8) $$h\leq \frac{G_{c}E}{10(1-v^{2})\sigma_{0}}$$

where *E* represents the Young’s modulus; *v* signifies Poisson’s ratio; and *G*_c_ is the critical fracture energy release rate at the crack tip. The element size in the crack extension region is 0.06 mm × 0.06 mm, which satisfied the demands of Equation (8).

### 3.4. Boundary Conditions and Loads

**Figure 4:**
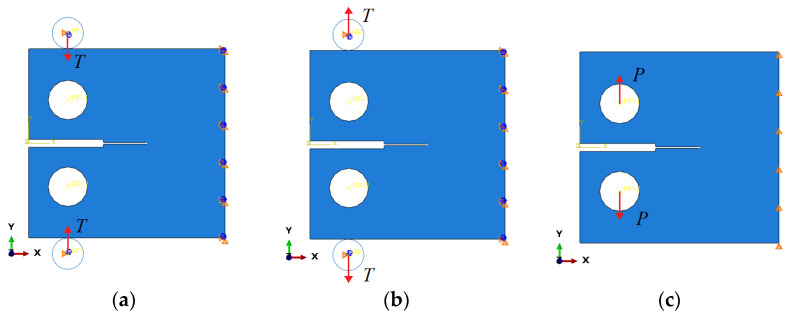
Three calculation steps: (**a**) pre-compression displacement loads are applied, (**b**) displacement loads are unloaded, and (**c**) external loads are applied to make the crack propagate.

The numerical calculation process is divided into three steps. In the first step, a specific compressive displacement *T* is applied to the rigid bodies at the upper and lower ends of the specimen, with constraints placed on the movement and rotation of the right side of the specimen. In the second step, the displacement load applied in the first step is released to obtain the RS field, and the boundary conditions remain unchanged. Finally, a displacement load of *P* = 1.5 mm is applied to each loading hole to initiate crack propagation. Rotation in the X-Y plane is permitted during the last calculation step. Figure 4 illustrates the boundary conditions and applied loads.

## 4. Crack Propagation in the Residual Stress Field

**Figure 5:**
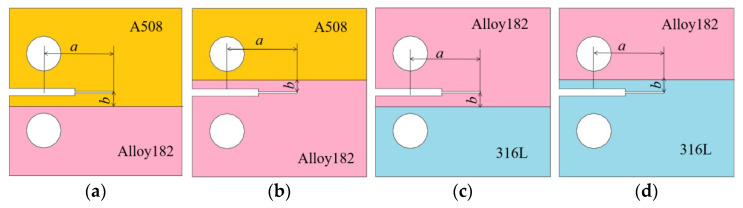
Locations of four cracks: (**a**) crack 1; (**b**) crack 2; (**c**) crack 3; and (**d**) crack 4.

In Figure 5, *a* represents the initial crack length and *b* denotes the distance from the fusion boundary (FB) line to the specimen’s symmetrical line. Four specimens are used to illustrate the sampling locations near the interface of two materials. Yellow represents A508, pink represents Alloy 182, and blue represents 316L. Crack 1 is situated in A508 near the interface of A508 and Alloy 182, crack 2 is located in Alloy 182 near the interface of Alloy 182 and A508, crack 3 is positioned in Alloy 182 near the interface of Alloy 182 and 316L, and crack 4 is found in 316L near the interface of Alloy 182 and 316L.

### 4.1. Variation of the Residual Stress around the Cack Front

**Figure 6:**
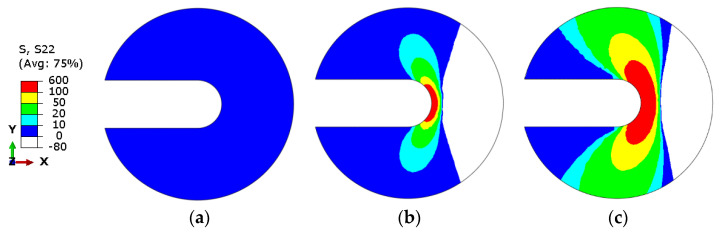
Residual stresses of crack 1 under different compressive displacements: (**a**) *T* = 0.03 mm; (**b**) *T* = 0.06 mm; and (**c**) *T* = 0.09 mm.

The variation of RS around the crack front under different compressive displacements is illustrated in Figure 6. The radius of the analyzed zone is 1 mm. There is almost no RS when the compressive displacement *T* is 0.03 mm, and the RS is on the verge of reaching the maximum tensile stress of the material when *T* equals to 0.09 mm. RS increases as the compressive displacement increases. Additionally, the RS around the crack front undergoes a gradual transition from tensile stress to compressive stress along the X-axis direction.

### 4.2. Effect of Residual Stress on Crack Propagation

**Figure 7:**
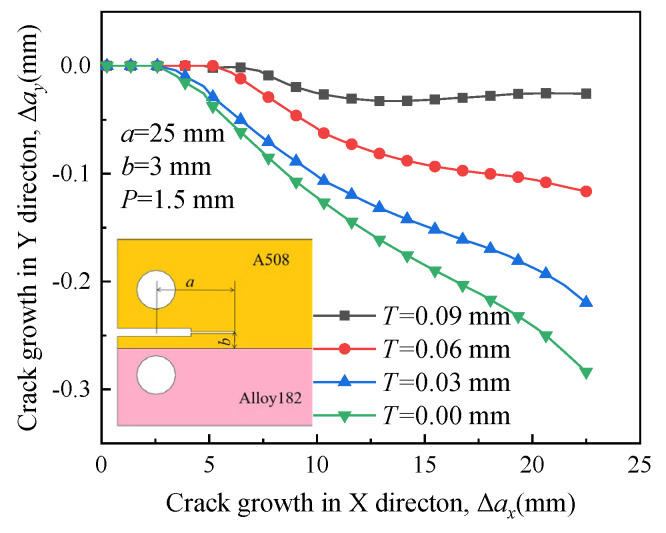
Propagation paths of crack 1 under different residual stress.

Figure 7 illustrates the propagation paths of crack 1 under different RS conditions. The crack propagates toward the boundary between Alloy 182 and A508 when there is no RS around the crack front. The crack propagation path will change with the distribution of RS around the crack front. The crack tends to grow in bulk A508 when the RS is very large, resulting in the propagation path of an approximately straight line. Therefore, the crack driven by RS exhibits a tendency to alter its growth direction.

**Figure 8:**
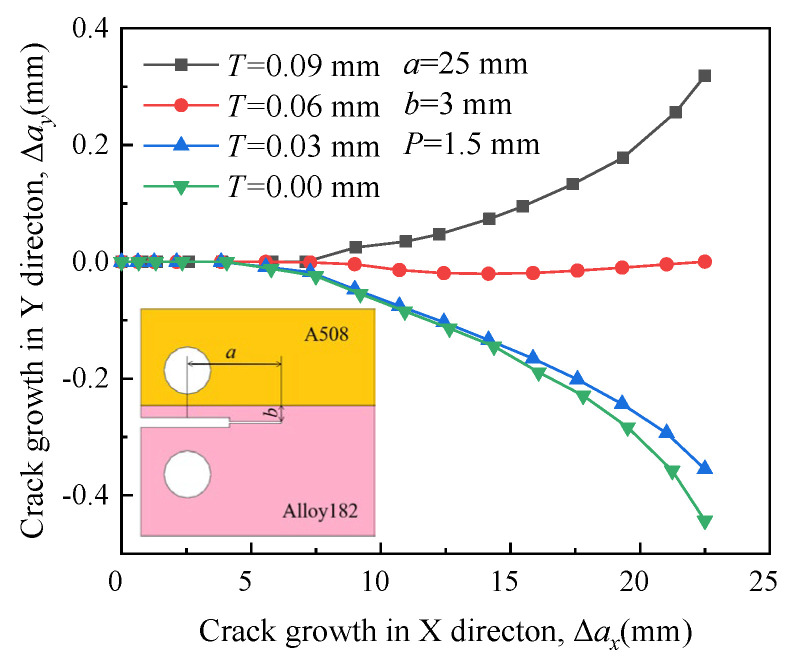
Propagation paths of crack 2 under different residual stress.

In Figure 8, the paths are similar when the RS is small. However, when the RS is very large (*T* = 0.09 mm), the path clearly deflects toward the boundary between A508 and Alloy 182. This observation suggests that the influence of RS on the propagation path is more significant than that of the material properties mismatch. Significantly high RS forces the crack to deviate from its initial direction into A508, which possesses a higher yield strength.

**Figure 9:**
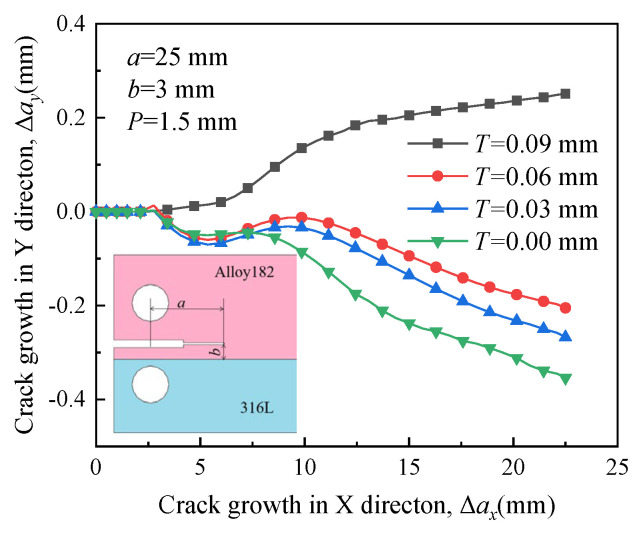
Propagation paths of crack 3 under different residual stress.

Figure 9 illustrates the influence of RS on the propagation paths of crack 3. When the RS is small, the crack propagates toward the boundary between Alloy 182 and 316L. However, as RS increases, its extension path deflects into the bulk of Alloy 182. In the presence of RS, the crack will deviate toward the material with higher yield strength.

**Figure 10:**
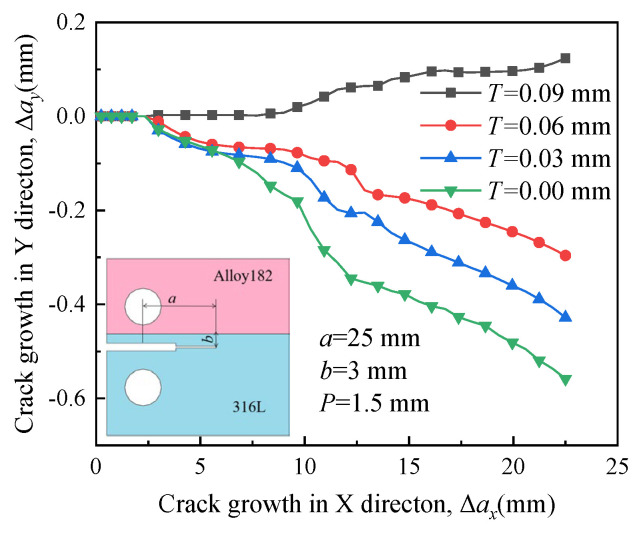
Propagation paths of crack 4 under different residual stress.

Figure 10 shows that the propagation paths of crack 4 in a DMW joint vary significantly under different RS conditions. When there is no RS present around the crack, it propagates into the bulk of 316L due to its location in a material with lower yield strength. Under the influence of RS, the direction of crack growth deflects toward the boundary between 316L and Alloy 182.

**Figure 11:**
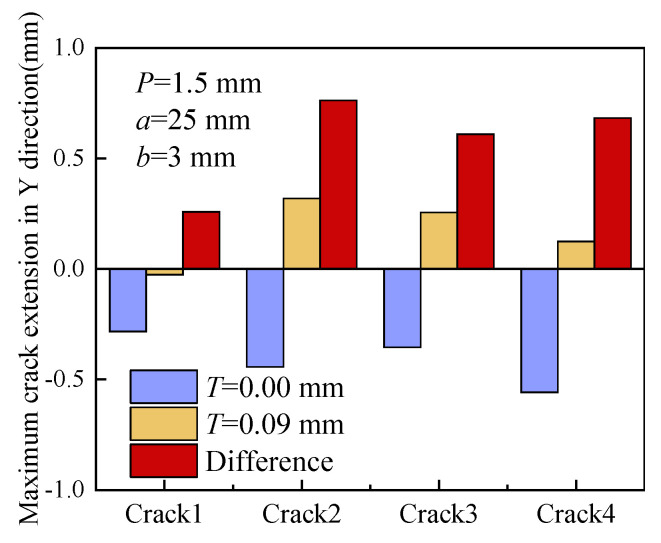
Maximum crack growth lengths in the Y direction.

Figure 11 illustrates the maximum crack growth lengths of the specimen in the Y direction. The purple and yellow rectangles indicate the maximum crack growth length when the displacement load is 0 mm and 0.09 mm, respectively. Additionally, the red rectangle indicates the difference in the maximum growth length between the two loads. With the absence of RS, all four cracks deflect toward the material with low yield strength. Crack 4, located in the bulk 316L, which has the lowest yield strength, exhibits the longest crack growth length in the Y direction.

When the introduced RS (*T* = 0.09 mm) approaches the material yield strength, cracks are more likely to extend toward the material with a high yield strength. This indicates that a large RS has a significant effect on the direction of crack growth. Crack 2 has the maximum crack growth length due to the largest yield strength mismatch between Alloy 182 and A508. Elevated RS results in the accumulation of high stress and high strain in the vicinity of the crack tip, which can increase the crack driving force to some extent. Material properties, such as fracture toughness and yield strength, can be treated as the resistant force to crack propagation. Actually, the deflection of the crack growing direction is the competition result between the crack driving force and the resistant force. High RS can change the stress state at the crack tip and ultimately change the competition result.

### 4.3. Effect of Young’s Modulus on Crack Propagation

The direction of crack extension can be influenced by the mechanical properties of a variety of materials; therefore, the crack extension curves were also analyzed when Young’s modulus was the same in both areas of the specimen, as well as when it was not. The values of Young’s modulus adopted in the simulations are shown in Table 2.

**Figure 12:**
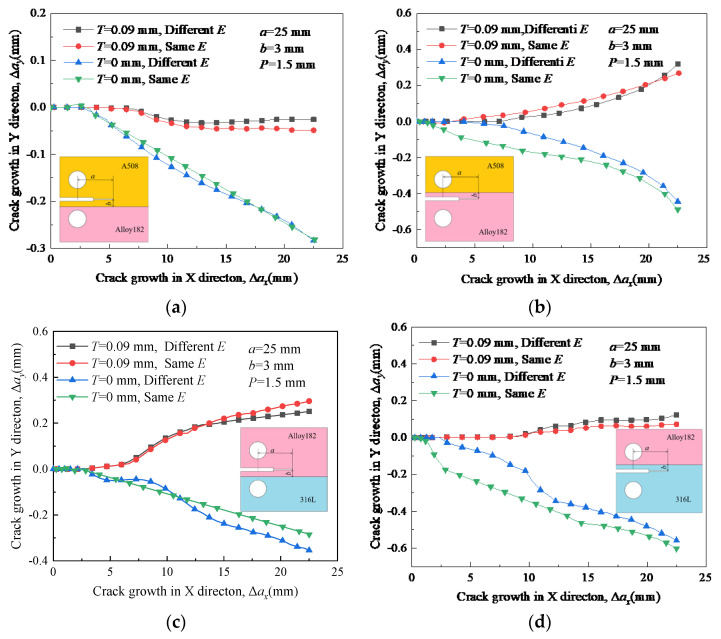
Propagation paths of cracks under different Young’s modulus: (**a**) crack 1; (**b**) crack 2, (**c**) crack 3; and (**d**) crack 4.

In Figure 12, the propagation path of crack 1 is minimally affected by Young’s modulus when there is no residual stress. The paths of crack 2 and crack 4 without RS fluctuate much more when the crack growth length is less than 15 mm. The reason is that crack 2 and crack 4 are both located in the material with low yield strength. When RS is introduced, four types of cracks all changed their original propagation directions toward the material that possesses a higher yield strength. Young’s modulus has relatively little impact on the crack propagation path. The maximum crack propagation length changes in the Y direction for the four types of cracks are 0.023 mm, 0.049 mm, 0.035 mm, and 0.051 mm, respectively. It indicates that Young’s modulus has less impact on the crack deflection than RS. The fluctuation of the crack propagation path with elevated RS is smaller than that without RS, but the changes are not significant.

### 4.4. Effect of Crack Location on Crack Propagation

**Figure 13:**
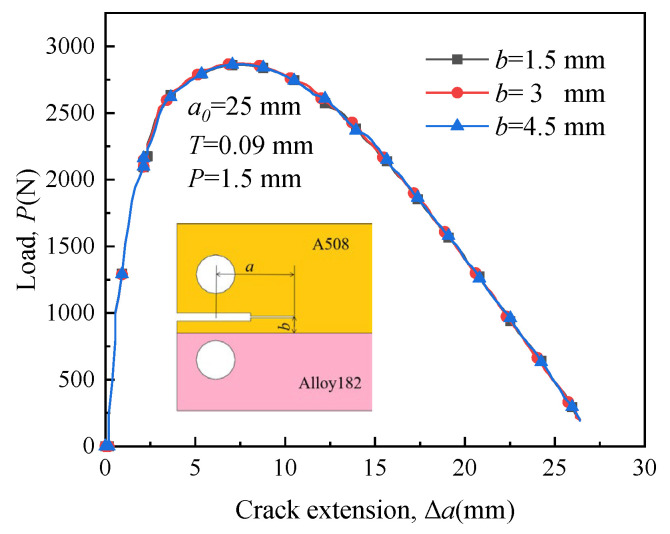
Load–length curves of crack 1 with different locations.

Figure 13 illustrates the variation of load required for crack growing when crack 1 is located at different positions. The initiation resistance of a long crack is smaller than that of a short crack. Long cracks tend to propagate more easily. The distance between the initial crack and the boundary line has little effect on crack initiation and propagation. The load variation pattern required during the crack propagation of crack 2, crack 3, and crack 4 is similar to that of crack 1; therefore, it is not described further.

**Figure 14:**
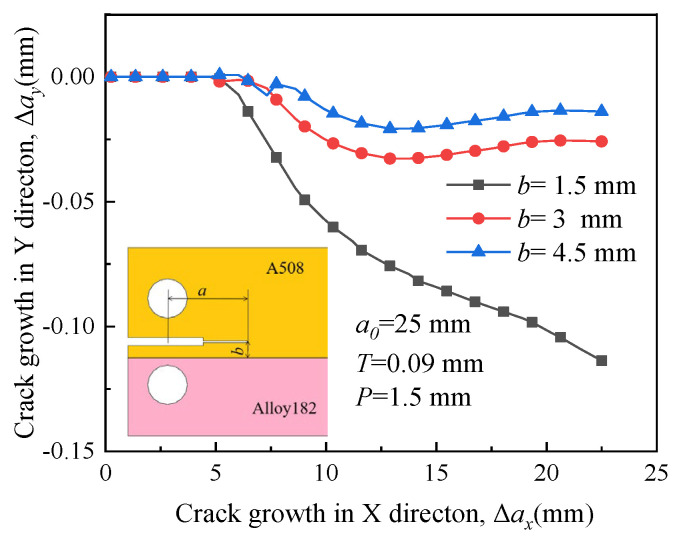
Propagation paths of crack 1 with different locations.

Figure 14 depicts the propagation paths of crack 1 at distances of 1.5 mm, 3 mm, and 4.5 mm between the specimen’s symmetric line and the boundary line. The initial crack length, denoted as *a*_0_, is 25 mm, and the compression displacement *T* is 0.09 mm. A displacement load of 1.5 mm is applied to drive crack growth. Due to the presence of a large RS, the crack tends to extend along the initial direction in A508, as shown in Figure 7, when the distance *b* is 3 mm and 4.5 mm. However, when the crack is situated adjacent to the interface of two materials (*b* = 1.5 mm), the crack extension deflects toward the material with low yield strength, indicating that the influence of RS on the direction of crack growth is not significant.

**Figure 15:**
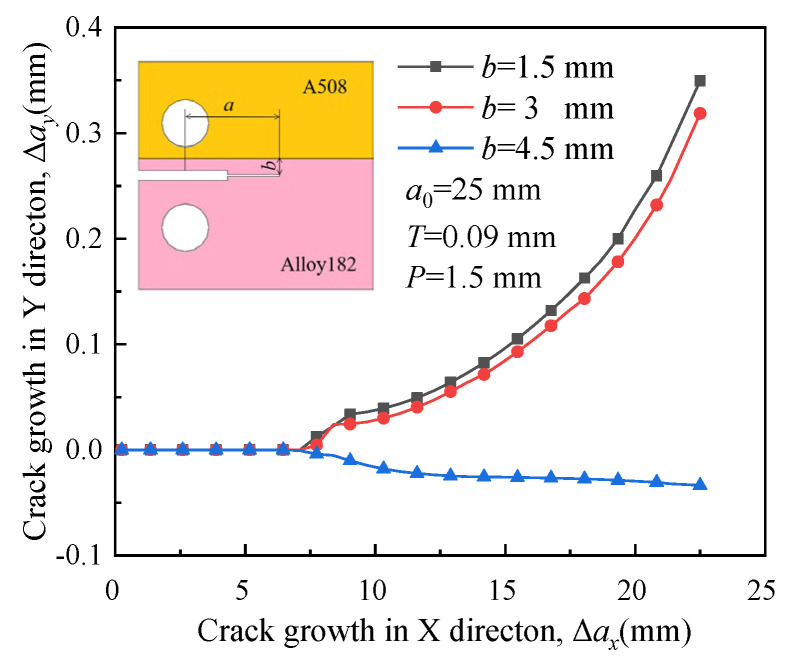
Propagation paths of crack 2 with different locations.

In Figure 15, the crack propagation path exhibits minimal fluctuation, and the crack tends to extend along the initial direction shown in Figure 8 when the distance is small (*b* = 1.5 mm and 3 mm). Hence, the influence of RS on the path is evident. However, when the initial crack is situated in a material with low yield strength and is far from the interface (*b* = 4.5 mm), RS has minimal impact on the direction of crack growth, and the crack propagates in Alloy 182.

**Figure 16:**
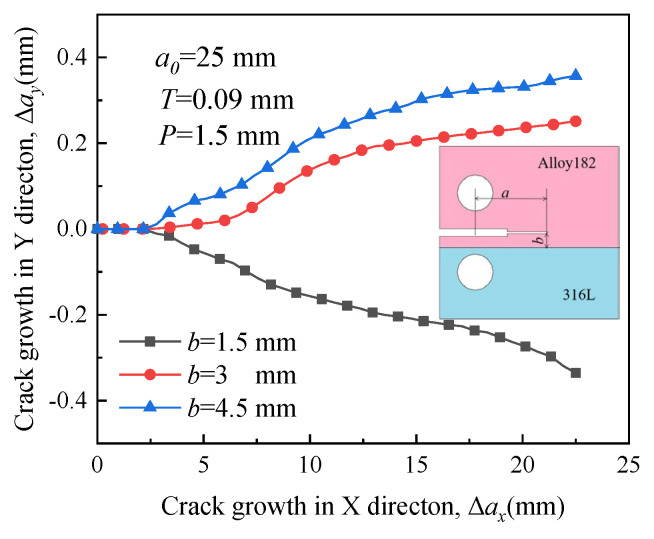
Propagation paths of crack 3 with different locations.

Figure 16 depicts the propagation paths of crack 3 in different positions. Similar to Figure 14, the crack tends to propagate along the initial direction (as shown in Figure 9) with an increase in *b*. RS has a significant effect on the crack propagation path when the initial crack is located far from the region with high yield strength material. When it is adjacent to the interface of Alloy 182 and 316L (*b* = 1.5 mm), the crack deflects toward the region with low yield strength because of no influence from RS.

**Figure 17:**
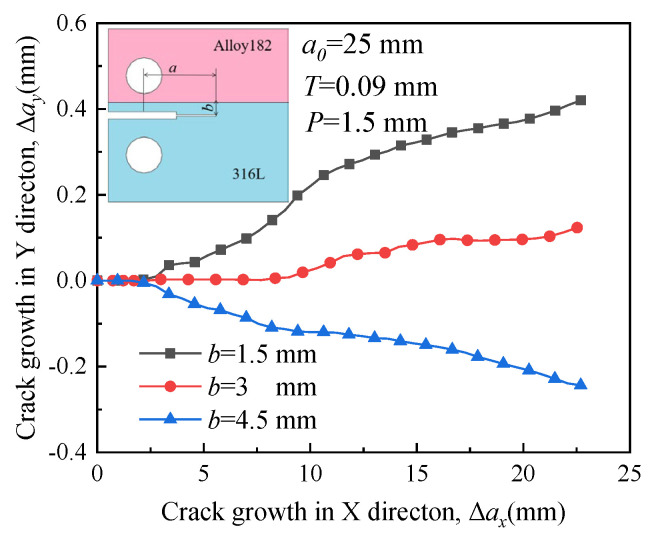
Propagation paths of crack 4 with different locations.

The propagation paths of crack 4 exhibit significant differences in Figure 17. Under the influence of RS, the crack propagates toward the material with high yield strength when the distance *b* is small. However, when the crack is far from the interface (*b* = 4.5 mm), the direction of crack growth is entirely different from the initial direction in Figure 10. This difference arises because the initial crack is located in a region with low yield strength, and the influence of RS on crack growth is not pronounced.

### 4.5. Effect of Initial Crack Length on Crack Propagation

**Figure 18:**
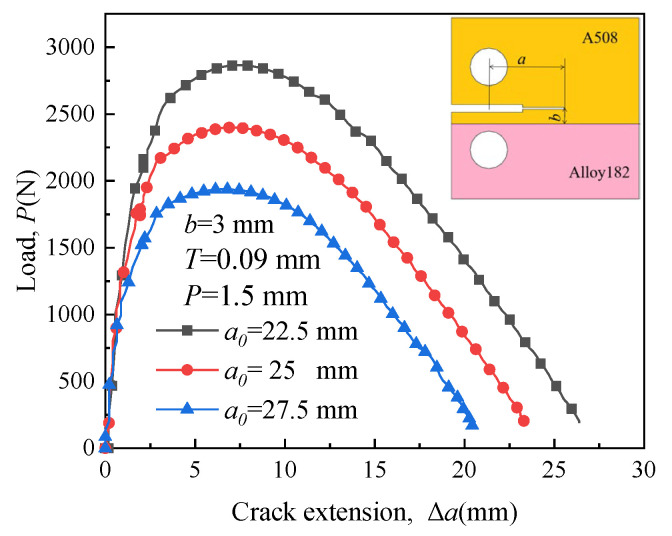
Load-length curves of crack 1 with different initial crack lengths.

Figure 18 illustrates the load–length curves of crack 1 with different initial crack lengths. The longer the initial crack length, the less load is required for crack growth. Therefore, long cracks tend to extend more easily. The patterns of load variation required for cracks 2, 3, and 4 are similar to those of crack 1.

**Figure 19:**
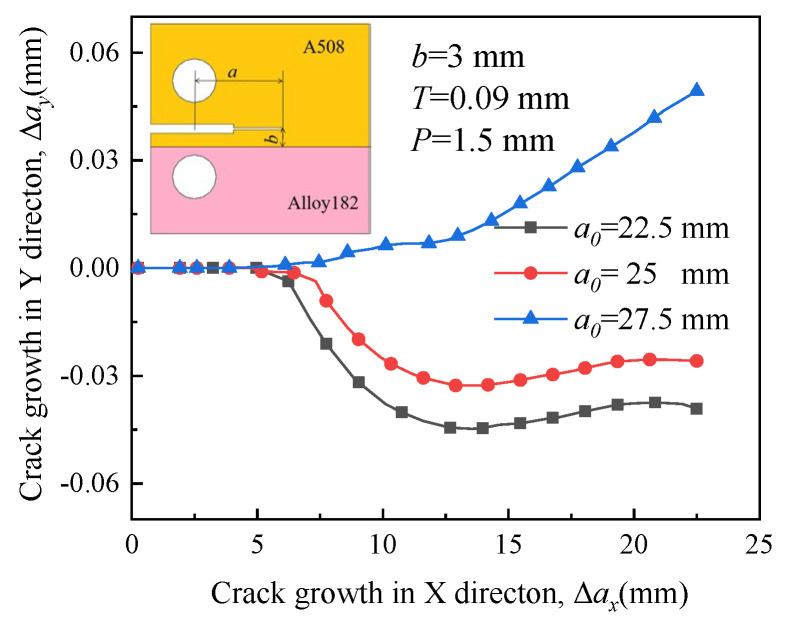
Propagation paths of crack 1 with different initial crack lengths.

**Figure 20:**
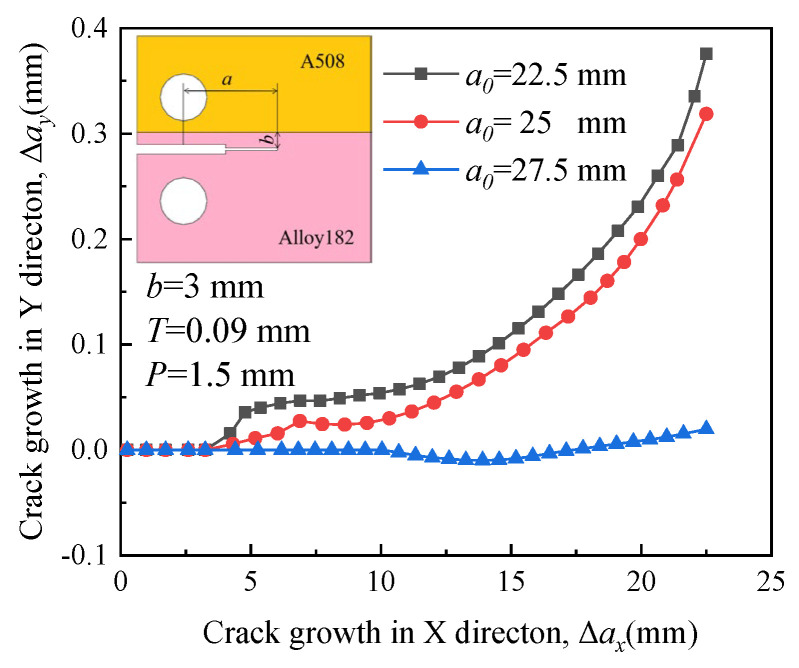
Propagation paths of crack 2 with different initial crack lengths.

**Figure 21:**
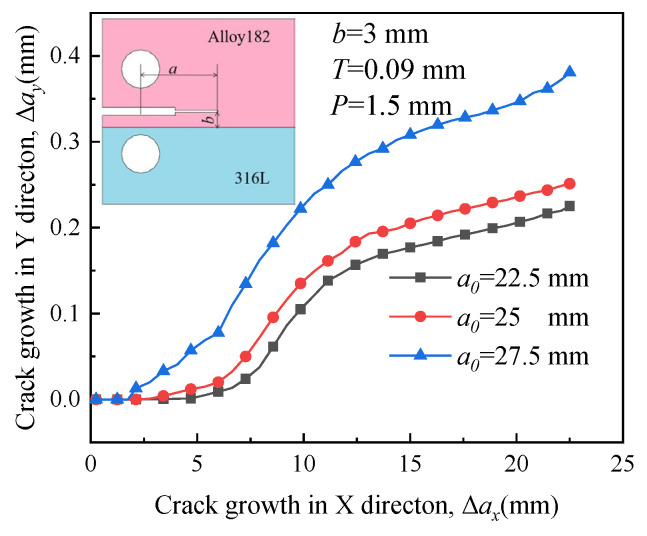
Propagation paths of crack 3 with different initial crack lengths.

**Figure 22:**
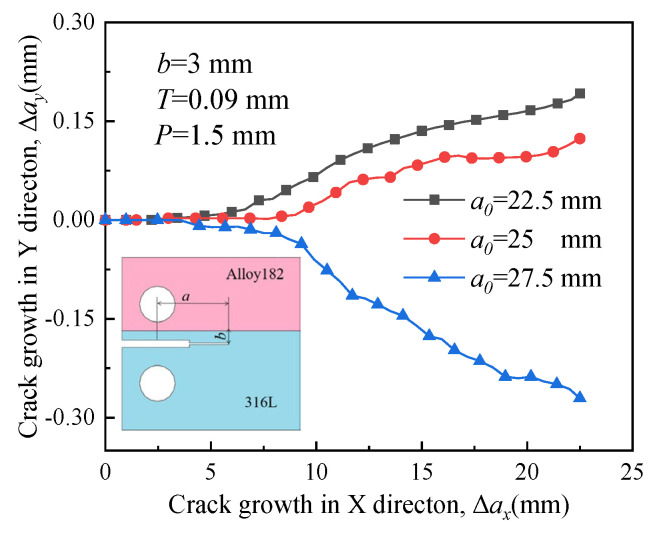
Propagation paths of crack 4 with different initial crack lengths.

Figure 19, Figure 20, Figure 21 and Figure 22 depict the crack propagation paths of four types of cracks with varying initial crack lengths. The initial crack lengths are 22.5 mm, 25 mm, and 27.5 mm. The distance between the specimen symmetric line and the boundary line *b* is 3 mm, and the compression displacement *T* is 0.09 mm. Under the influence of the applied load, denoted as *P* = 1.5 mm, the crack begins to extend.

In Figure 19 and Figure 21, driven by a large RS, a long initial crack (*a*_0_ = 27.5 mm) tends to extend toward the material with higher yield strength when it is located in the material with higher yield strength. This indicates that the influence of RS on the crack propagation path is significant. The direction of growth for a short crack deflects toward the material with low yield strength and is almost unaffected by RS. Crack deviation into the lower strength plate was also reported for laser beam welded dissimilar steel joints [38].

In Figure 20 and Figure 22, when the crack is situated in a material with lower yield strength, RS encourages the short initial crack (*a*_0_ = 22.5 mm and 25 mm) to extend toward the material with high yield strength. The growth direction of a long initial crack deflects toward the material with low yield strength, indicating that the influence of RS on the crack propagation path is not significant.

## 5. Conclusions

Based on XFEM technology, the propagation paths of cracks in DMW joints under different RS states were predicted and compared. The effects of RS, Young’s modulus, crack location, and initial crack length on crack propagation were investigated. Propagation paths of cracks in DMW joints under various RS states were predicted and compared. The crack growing direction in a DMW joint without RS deflects to the material with low yield strength. The crack initiation and propagation within the RS field retain almost no effect from the distance between the specimen’s symmetric line and the boundary line. Nevertheless, elevated RS significantly urges the crack growing direction to deflect toward the material of high yield strength. Young’s modulus has little impact on the deflection of crack propagation paths. A short crack has less resistance to crack initiation than a long crack, and a long crack is more prone to initiate and extend. For long cracks and those located far from the interface of two materials, the influence of RS on the crack propagation path is significant when they are situated in materials with high yield strength. The crack growing direction deflects to the high yield strength side of the material. However, if the initial crack is located in a material with low yield strength, elevated RS prompts a short crack, which is adjacent to the interface of two materials, to propagate toward the high yield strength side.

## Figures and Tables

**Table 1:** Pertinent constitutive parameters of materials in a DWM joint [14,35,36].

Materials	Young’s Modulus *E* (GPa)	Poison’s Ratio *v*	Yield Stress *σ*_0_ (MPa)	Hardening Exponent *n*	Ultimate Tensile Strength *σ*_u_ (MPa)	Fracture Toughness *J_IC_* (kJ/m^2^)
A508	183	0.3	385	4.779	560	224.2
Alloy 182	203	0.3	342	4.375	576	166.6
316L	176	0.3	309	4.080	485	170.0

**Table 2:** Young’s modulus adopted in the simulations.

Crack Location	Different Young’s Modulus *E* (GPa)	Same Young’s Modulus *E* (GPa)
crack 1	*E*_A508_ = 183, *E*_Alloy 182_ = 203	*E*_Alloy 182_ = 203
crack 2		
crack 3	*E*_Alloy 182_ = 203, *E*_316L_ = 176	
crack 4		

## Data Availability

The data supporting the findings of this study were calculated by using XFEM and are presented in this article. The parameters employed in the model were referenced from the listed sources.
